# Iron Supplement-Enhanced Growth and Development of *Hydrangea macrophylla* In Vitro under Normal and High pH

**DOI:** 10.3390/cells10113151

**Published:** 2021-11-13

**Authors:** Jie Xiao, Ge Guo, Byoung Ryong Jeong

**Affiliations:** 1 Department of Horticulture, Division of Applied Life Science (BK21 Four), Graduate School of Gyeongsang National University, Jinju 52828, Korea; xiaojsicau@163.com (J.X.); rainbowmaomao317@gmail.com (G.G.); 2 Institute of Agriculture and Life Science, Gyeongsang National University, Jinju 52828, Korea; 3 Research Institute of Life Science, Gyeongsang National University, Jinju 52828, Korea

**Keywords:** antioxidant system, chlorophyll, chlorosis, photosynthesis, plant reproduction, thylakoid

## Abstract

*Hydrangea macrophylla* is a popular perennial ornamental shrub commercially grown as potted plants, landscape plants, and cut flowers. In the process of reproduction and production of ornamental plants, the absorption of nutrients directly determines the value of the ornamental plants. *Hydrangea macrophylla* is very sensitive to the content and absorption of the micronutrient iron (Fe) that affects growth of its shoots. However, the physiological activity of Fe as affected by deficiency or supplementation is unknown. This work aimed at preliminary exploring the relationship between Fe and photosynthesis, and also to find the most favorable iron source and level of pH for the growth of *H. macrophylla*. Two Fe sources, non-chelated iron sulfate (FeSO_4_) and iron ethylenediaminetetraacetic acid (Fe-EDTA), were supplemented to the multipurpose medium with a final Fe concentration of 2.78 mg·L^−1^. The medium without any Fe supplementation was used as the control. The pH of the agar-solidified medium was adjusted to either 4.70, 5.70, or 6.70, before autoclaving. The experiment was conducted in a culture room for 60 days with 25/18 °C day and night temperatures, and a 16-hour photoperiod provided at a light intensity of 50 mmol·m^−2^·s^−1^ photosynthetic photon flux density (PPFD) from white light-emitting diodes. Supplementary Fe increased the tissue Fe content, and leaves were greener with the medium pH of 4.70, regardless of the Fe source. Compared to the control, the number of leaves for plantlets treated with FeSO_4_ and Fe-EDTA were 2.0 and 1.5 times greater, respectively. The chlorophyll, macronutrient, and micronutrient contents were the greatest with Fe-EDTA at pH 4.70. Furthermore, the Fe in the leaf affected the photosynthesis by regulating stomata development, pigment content, and antioxidant system, and also by adjusting the expression of genes related to Fe absorption, transport, and redistribution. Supplementation of Fe in a form chelated with EDTA along with a medium pH of 4.70 was found to be the best for the growth and development of *H. macrophylla* plantlets cultured in vitro.

## 1. Introduction

*Hydrangea macrophylla* is a popular perennial summer-flowering shrub with an enormous inflorescence and top growth. It is one of the most promising ornamental flower species for use as potted plants, landscape plants, and cut flowers [1,2]. It originates from eastern Asia and North America, and is widely planted in the temperate regions and extends southward into the tropics of both hemispheres [3]. The *H. macrophylla* extract is effective against the human malaria parasite, diabetes, and urinary tract infection [4,5,6]. Furthermore, its root bark has been used to treat numerous kidney diseases [7]. Commercially, *H. macrophylla* is propagated either by seeds or stem cuttings. Although seed germination is not difficult, the variability of seedlings is high, and therefore seed propagation does not always lead to the desired characteristics and morphology for the producer. On the other hand, cutting propagation is slow to establish, and plants from cuttings lack good basal branching [8,9]. Micropropagation is widely used in ornamental horticulture. However, it is limited for many in vitro perennial trees and vegetative shrubs, and few studies have been conducted with in vitro *H. macrophylla* [10].

Iron (Fe) participates in photosynthesis, mitochondrial respiration, nitrogen assimilation, hormone (ethylene, gibberellic acid, jasmonic acid, etc.) biosynthesis, production and scavenging of reactive oxygen species, osmoprotection, and pathogen defense [11]. Moreover, Fe influences the formation of coproporphyrin in the tetrapyrrole biosynthetic pathway and is also necessary for chlorophyll synthesis [12]. The uptake, distribution, and storage of Fe are tightly regulated in plants [13]. There are two different responses of plant roots induced by Fe deficiency. Dicotyledons and non-gramineous plants utilize the strategy I. Firstly, the activity of a plasma membrane-bound reductase in the root increases when Fe is deficient, which enhances the rate of Fe III reduction and correspondingly splits Fe-III-chelates at the plasma membrane by increasing the acidification of the rhizosphere. Meanwhile, roots produce ferric chelate reductase (FCR) to catalyze Fe^3+^ to Fe^2+^ [14]. Then the iron regulated transporter 1 (*IRT1*) transports the Fe^2+^ into the cells of the root. Simply, it is an acidification-reduction-transport mechanism, and proton pump H^+^-ATPase 2 (AHA2), ferric chelate reductase 2 (*FRO*2), and IRT1 can assemble an iron-acquisition complex to optimize Fe uptake during this progress [15]. The expression of these three transporters is regulated by bHLH transcription factors, *FER-like iron deficiency induced transcription factor* 1 (*FIT1*), which is the homolog gene of *ferritin* (*FER*) can regulate the accumulation of *FRO2* and *IRT1* [16]. Gramineae plants utilize a different strategy (strategy II) based on chelation, they can produce the mugineic acid family phytosiderophores (MAs) to complex sparingly soluble inorganic Fe^3+^ The iron-related bHLH transcription factor 2 (*IRO2*) has a key role to regulate the expression of MAs biosynthetic genes [17]. Then the TOM1 transports the MAs outflow to the rhizosphere. Finally, the Fe-MAs complexes enter the root by the yellow stripe-like 1 (YSL1) [18]. Nonetheless, these two strategies are similar; for example, rice can use either strategy I or II according to the concentration of substrate Fe [17]. The metabolites of the partial strategy I plants have the characteristic of strategy II plants [19]. Moreover, the transportation of Fe is dependent on the nicotianamine (NA) both in strategy I and strategy II in the symplast [20]. Although the molecular mechanism of Fe-deficiency responses operating in roots is well documented, the genes controlling these activities in shoots are unknown. The transportation of Fe is not only located in the root but also regulated by the whole plant. For instance, the expressions of *IRT1* and *FEO2* genes and accumulation of MAs protein increased during the day and down-regulated at night, however, accumulation increased during the night when the plant suffered Fe-deficiency. Besides, Fe transport is regulated by the signals of shoot-borne and nutrients such as sugar [21]. In *Arabidopsis*, many of the regulators which have been identified in roots were also found in leaves under Fe deficiency, including the photosynthesis-related genes photosystem I subunit F (*PsAF)* and photosystem I subunit N (*PsAN*), ferredoxin (*FED*), and proteins family light-harvesting complexes-related (LHCB). However, there is little information about the inference of Fe signal transduction in chloroplast and leaf [22].

It is clear that the effects of substrates on Fe absorption and transport, all related genes of strategy I were decreased at pH 7.5 [23], and a high concentration of HCO^3−^ limits the rate of Fe uptake due to the solubility of Fe decreases 1000-fold for each unit increase of pH. [24]. Therefore, local acidification of calcareous soils is found to be an effective, inexpensive means to alleviate iron chlorosis [25]. Besides, injecting iron solution into the trunks of trees or the surrounding soils, and mixing iron into the root systems at the transplanting time are corrective procedures for Fe deficiency. However, different plants have different sensitivities or tolerances to iron deficiency. For acidophilus plants, the most obvious effect of Fe deficiency is the youngest leaves turning yellow and stunt plant growth due to the decrease in photosynthetic efficiency and electron transport through PSII [13]. Interestingly, the activity of PSI in chlorosis leaves was only slightly inhibited under Fe deficiency [26]. Besides, at the ultrastructural level, although the thylakoid system markedly decreases, other Fe-containing organelles as mitochondria and peroxisomes are immune [27]. One view is that the Fe deficiency induces a large xanthophyll cycle. This cycle is beneficial to photoprotection and reduces the injury by increasing leaves oxygen concentration [28,29]. However, another view is the xanthophyll cycle and the resynthesis of photosynthetic pigments in Fe deficiency plants occur after Fe resupply [30]. Therefore, the effect of Fe on photosynthesis and even for the physiological activities of the whole plant is still incomplete.

The *H. macrophylla* is susceptible and slowly dies in response to Fe deficiency. However, the physiological activity of Fe as affected by deficiency or supplementation is unknown. It is necessary to investigate the responses to different Fe sources and pH levels. In our previous studies, supplemental FeSO_4_ or Fe-EDTA have been found to have a growth-promoting effect on sensitive plants *Sorbus commixta* [31]. However, the regulation mode of Fe is not discussed in depth. In this study, the photosynthesis and the content of the pigment of leaves were measured, and the macro- and micronutrients in leaves were analyzed. Lastly, we studied the relative expressions of Fe transfer and photosynthesis genes, to dissect the response of *H. macrophylla* Fe deficiency, and preliminary explore the relationship between Fe nutrient and photosynthesis, and also to find the optimal combination for alleviating chlorosis for *H*. *macrophylla.*

## 2. Materials and Methods

### 2.1. Plant Materials and Culture Conditions

Excised apical and axillary buds of young *H. macrophylla* branches from a glasshouse at Gyeongsang National University (GNU), Jinju, Korea (35°09′ N, 128°05′ E) were used. The explants were washed under running tap water for 1 h, rinsed thoroughly with distilled water, and subsequently disinfected in a 70% (*v*/*v*) ethanol solution for 30 s, a 1.5% (*v*/*v*) sodium hypochlorite solution with a drop of Tween 20 for 10 min, and each explant was finally washed 4 times with sterile distilled water. The explants were placed in a culture container (MB-G0202, Kisan Bio Co., Ltd., Seoul, Korea) containing 50 mL of the MS [32] medium with 3.0% sucrose (*w*/*v*) and 0.8% agar (*w*/*v*), supplemented with 0.2 mg·L^−1^ 6-benzyl amino purine. The explants were maintained in a growth chamber for 30 days. Roots were induced on the half-strength MS medium with 0.25 mg·L^−1^ l-naphthyl acetic acid for 30 days. Newly cut buds and shoots were transferred to the multiplication medium without any PGRs and sub-cultured at 30-day intervals. All cultures were maintained at 25 °C and with a 16-hour photoperiod provided by cool white fluorescent light (40 W tubes, Philips) at an intensity of 50 mmol·m^−2^·s^−1^ photosynthetic photon flux density (PPFD).

### 2.2. Iron and pH Treatments

The 2.0 cm-long singularized shoots were excised from multiple shoots and cultured on the multipurpose (MNS) medium. This nutrient solution was made from Ca(NO_3_)_2_·4H_2_O 467.6, KNO_3_ 232.3, KH_2_PO_4_ 272.0, K_2_SO_4_ 17.4, MgSO_4_·H_2_O 209.1, NH_4_NO_3_ 80.0, H_3_BO_3_ 1.4, NaMoO_4_·2H_2_O 0.12, MnSO_4_·4H_2_O 2.10, ZnSO_4_·7H_2_O 0.80, and CuSO_4_·5H_2_O 0.20 (mg·L^−1^), and contained 3.0% sucrose (*w*/*v*) and 0.8% agar (*w*/*v*). For Fe sources, non-chelated iron sulfate (FeSO_4_), or iron ethylenediaminetetraacetic acid (Fe-EDTA) were added to the multipurpose medium with a final Fe concentration of 2.78 mg·L^−1^. The medium for the control had no supplementary Fe. The pH of the agar-solidified medium was adjusted to either 4.70, 5.70, or 6.70 using 1 M NaOH or HCl before autoclaving.

### 2.3. Measurement of the Growth Characteristics

After 60 days, plant parameters—the number of roots and leaves, the shoot, root, and stem lengths, leaf area, leaf color, leaf fresh and dry weights—were examined. The fresh weight was measured with an electronic scale (EW 220-3NM, Kern and Sohn GmbH., Balingen, Germany). Samples were dried using a forced air-dry oven (Venticell-222, MMM Medcenter Einrichtungen GmbH., Munich, Germany) at 70 °C for 72 h before the dry weights were recorded. The leaf area was measured with a leaf area meter (LI-3000, LI-COR Inc., Lincoln, NE, USA). The color values of the leaves were measured with a color reader CR-11 (1994 Minolta Co., Ltd. Osaka, Japan). The photosynthesis was measured with a FluorPen FP 100 (Photon Systems Instruments, PSI, Drásov, Czech Republic).

### 2.4. Contents of Chlorophyll, Carotenoid, and Total Anthocyanin

To estimate the chlorophyll content, 100 mg leaf tissues were submerged in acetone for 12 h to form the extraction solution. The absorbances of the liquid supernatant were measured at 645 and 663 nm with a UV-spectrophotometer (Libra S22, Biochrom Ltd., Cambridge, UK). The contents of chlorophyll a and b, and carotenoid were determined using the following formulae [33]:

(1) $$\mathrm{Chlorophyll}\;a=\frac{\left(11.75\;\times \;\mathrm{OD}\;\mathrm{at}\;663\;\mathrm{nm}\;–\;2.35\;\times \;\mathrm{OD}\;\mathrm{at}\;645\;\mathrm{nm}\right)\;\times \;V*}{\mathrm{Sample}\;\mathrm{fresh}\;\mathrm{weight}}$$

(2) $$\mathrm{Chlorophyll}\;b=\frac{\left(27.05\;\times \;\mathrm{OD}\;\mathrm{at}\;645\;\mathrm{nm}\;–\;11.21\;\times \;\mathrm{OD}\;\mathrm{at}\;663\;\mathrm{nm}\right)\;\times \;V}{\mathrm{Sample}\;\mathrm{fresh}\;\mathrm{weight}}$$

(3) $$\mathrm{Carotenoid}=\frac{1000\;\times \;\mathrm{OD}\;\mathrm{at}\;470\;\mathrm{nm}\;–\;2.27\;\times \;\mathrm{Chlorophyll}\;a\;-\;81.4\;\times \;\mathrm{Chlorophyll}\;b}{227}$$

(*V, the volume of the extraction solution. The pigment content was expressed as mg of chlorophyll a and b, and carotenoid per g of fresh leaf weight).

The total anthocyanin content was measured using a UV spectrophotometer [34]. Briefly, the leaf tissue (0.5 g) was extracted with 2 mL of 1% HCl-ethanol and centrifuged (Centrifuge 5428, Eppendorf, Hamburg, Germany) at 13,000 rpm for 20 min at 4 °C. The extracts were acidified to approximately pH 1.0 using 0.1 N HCl, and the absorbance was subsequently measured at 530 nm.

### 2.5. Total Soluble Proteins and Antioxidant Enzyme Activities

The 100-mg leaf samples were homogenized in a 1.5-mL ice-cold 50-mM phosphate buffer (pH 7.0) containing 1 mM of ethylenediaminetetraacetic acid (EDTA), 0.05% Triton X-100, and 1 mM of polyvinylpyrrolidone (PVP). The extracts were centrifuged at 13,000 rpm for 20 min at 4 °C, and the supernatant was used immediately to determine the soluble protein contents and antioxidant enzyme activities. The soluble protein content and the activities of superoxide dismutase (SOD), ascorbate peroxidase (APX), catalase (CAT), and peroxidase (POD) were measured according to the established protocols of Soundararajan et al. [35,36].

### 2.6. FCR Activity

The FCR activity measurements were taken based on the method of Bienfait [37]. The composition of the reaction solution was as follows (in mol·L^−1^): 1 × 10^−6^ Fe (Ш)EDTA, 4 × 40^−4^ 2,2’-bipyridyl, 7.5 × 10^−4^ K_2_SO_4_, 6.5 × 10^−4^ MgSO_4_, 2.5 × 10^−4^ K_2_HPO_4_, 1 × 10^−3^ KCl, 1 × 10^−4^ H_3_BO_3_, 1 × 10^−6^ MnSO_4_, 5 × 10^−7^ CuSO_4_, 1 × 10^−6^ ZnSO_4_, 5 × 10^−8^ (NH_4_)_6_Mo_7_O_24_. Plants were placed with their roots in a 20-mL saturated CaSO_4_ solution for 5 min, and the roots were washed three times with distilled water, and soaked for 20 min in the reaction solution (pH 5.3) in the dark. The absorbance was subsequently measured at 520 nm. The FCR activity was determined using the following formula:

$$\mathrm{The}\;\mathrm{FCR}\;\mathrm{activity}=\frac{\left(\mathrm{OD}\;\mathrm{at}\;520\;\mathrm{nm}\right)\;\times \;10^{6}}{\mathrm{Root}\;\mathrm{fresh}\;\mathrm{weight}\;\times \;8650}$$

### 2.7. Scanning Electron Microscopic (SEM) Analysis of the Stomata

Leaf samples were cut into 0.5 mm^2^ pieces and fixed in 3.0% (*v*/*v*) glutaraldehyde (pH 7.5) at 4 °C overnight. Staining was done in a 1.0% osmium tetroxide solution for 2 h at 4 °C, and the samples were subsequently dehydrated in a graded series of 20, 40, 60, 80, 100% ethanol, and were finally immersed in 80% acetone. Dried samples were positioned on aluminum stubs with double-stick tape before being gold-coated in a sputter coater (SC7640; Polaron, Sussex, UK). A field emission scanning electron microscope II (SEM/EDS, JSM-7610F, JEOL Ltd., Tokyo, Japan) was used to observe the stomata [38].

### 2.8. Determination of Macro- and Micronutrient Contents Using Inductively Coupled Plasma Spectrometer

The macro- and micronutrient contents were measured according to the method of Zhang [39]. Briefly, leaves dried in a forced air-dry oven at 60 °C were finely powdered, and then 500 mg dry samples were ashed in a Nabertherm muffle furnace (Model LV 5/11/B180, Lilienthal, Breman, Germany) at 525 °C for 4 h. The ash was dissolved in 5 mL 25% HCl, and subsequently diluted with 15 mL of warm distilled water and 10 mL of room-temperature distilled water. The macro- and micronutrient contents were measured using an inductively coupled plasma (ICP) spectrometer (Optima 4300DV/5300DV, Perkin Elmer, Germany).

### 2.9. Quantitative Real-Time PCR Analysis

**Figure 1:**
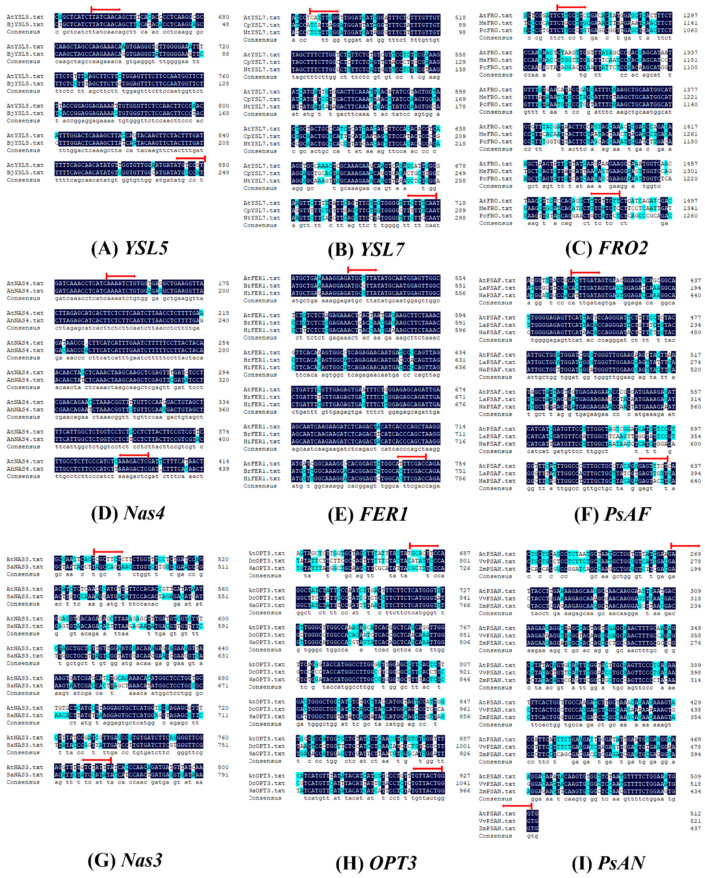
DNA sequence alignment diagrams for *AtYSL5* and *BjYSL5* (**A**); *AtYSL7*, *CpYSL7*, and *NtYSL7* (**B**); *AtFOR2*, *MeFRO2*, and *PcFRO2* (**C**); *AtNAS4* and *AhNAS4* (**D**); *AtFER1*, *BrFER1*, and *HiFER1* (**E**); *AtPsAF*, *LaPsAF*, and *NtPsAF* (**F**); *AtNAS3* and *SaNAS3* (**G**); *AtOPT3*, *DcOPT3*, and *NtOPT3* (**H**); *AtPsAN*, *VvPsAN*, and *ZmPsAN* (**I**). The alignment was obtained by using the DNAMAN software.

The CTAB method was adopted for the total RNA extraction [40]. The quality of the RNAs was determined with the NanoDrop 2000c Spectrophotometer (Thermo Fisher Scientific, Waltham, MA, USA), then reverse transcribed to cDNA using the PrimeScript RT Reagent Kit (Takara, Shiga, Japan). A total of 20 µL of reaction volume was constructed with 2 µL of each of the forward and reverse primers, 2 µL of cDNA, 10 µL of SYBR green, and 4 µL of RNase-free water. The DNA sequence blast with *Arabidopsis thaliana* was used, since the *H. macrophylla* genome has not yet been completely sequenced, along with the target genes from the following species, *Brassica juncea; YSL7, Cucurbita pepo,* and *Nicotiana tobacco; FOR, Cucurbita pepo,* and *Nicotiana tobacco; FOR, Arabidopsis helleri; FER1, Brassica rapa,* and *Hirschfeldia incana; PsAF, N. tobacco; NAS3, Sedum alfredii; OPT3, Dendrobium candidum,* and *N. tobacco;* and *PsAN, Vitis vinifera,* and *Zea mays.* The CDS sequences of *A. thaliana* (Table 1) found on The Arabidopsis Information Resource: ‘https://www.arabidopsis.org/’ (accessed on 9 July 2021) and others found on the National Center for Biotechnology Information: ‘https://www.ncbi.nlm.nih.gov/’ (accessed on 9 July 2021) were also used. The alignment was obtained (Figure 1) by using the DNAMAN software. After blasting against *Arabidopsis*, the target genes were chosen to determine their gene expression levels in this experiment. The primers used in this study are shown in Table 2. The running procedure was set to 95 °C for 3 min, followed by 40 cycles at 95 °C for 30 s, and 55 °C for 30 s used on the CFX96 real-time PCR system (Bio-Rad, Hercules, CA, USA). Three biological replicates were adopted for each treatment. The relative expression levels were calculated using the 2^−∆∆Ct^ method, using the control at pH 4.70 as a reference (value = 1).

### 2.10. Data Collection and Analysis

The statistical analysis was carried out using the Statistical Analysis Program (SAS 9.1, SAS Institute Inc., Cary, NC, USA). The experimental results were subjected to an analysis of variance (ANOVA) (*p* ≤ 0.05) and Duncan’s multiple range test (*p* ≤ 0.05). An F-test was also performed based on Fisher’s least significant difference test at a threshold of *p* = 0.05. The calculation code is as follows:

Data KYJ; infile ’location.prn’; input trt rep measurements; proc print; run; proc anova; class trt; model measurements = trt; means trt/duncan; title kyj; run; (the Duncan’s multiple range test).

Data KYJ; infile ’location.prn’; input A B repmeasurements; proc print; run; proc anova; class A B; model measurements = A B A*B; means A B A*B/lsd; title KYJ; run; (F-test).

Pearson’s correlation coefficient was calculated with the SPSS 17.0 software (SPSS Inc., Chicago, IL, USA). Click Analyze > Correlate > Bivariate. Select the variables and move them to the variables box. Graphing was performed with the OriginPro software (version 9.0).

## 3. Results

### 3.1. Analysis of the Morphological and Growth Parameters

**Figure 2:**
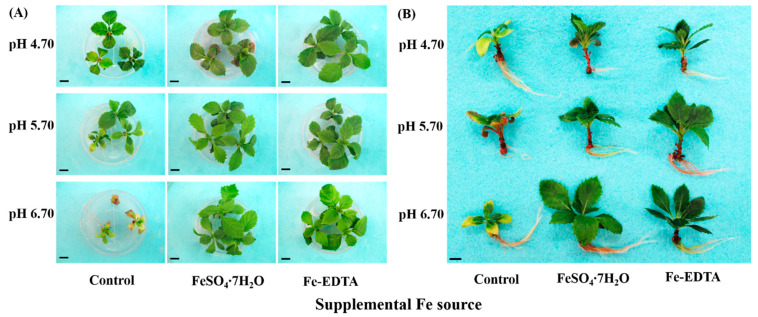
Photographs showing the morphology of *H. macrophylla* as affected by the Fe source and medium pH after 60 days of culture: (**A**) a top view of the plantlets, still in the medium; and (**B**) representative individual plantlets. The bars in each photograph indicate 1 cm.

After the 60-day treatments, the growth of *H. macrophylla* showed characteristic symptoms according to the Fe source and medium pH (Figure 2A,B). The plantlets in treatments without Fe sources were visibly more chlorotic and their growth was stunted, especially under high pH. The growth of plantlets in treatments with supplementary Fe appeared more robust than that of control, and the leaves were better developed (Table 3). Moreover, the plantlets grown under supplemental FeSO_4_ at pH 4.70 had the longest leaf and highest number of leaves. Nonetheless, the root growth were same within supplemental FeSO_4_ regardless of pH (Figure 2 and Table 3). These results of growth parameters expressed the effects of iron sources and pH on the growth potential of *H. macrophylla*.

### 3.2. Leaf Color and Pigment Content Analyses

Leaf color reflects the contents of pigment in leaves. The measured color values of *H. macrophylla* based on the CIELAB system are given in Table 4. In the evaluation of the color brightness, (*L*) means ‘Lightness’ and ranges from ‘0’ (darkness) to ‘100’ (white). The color coordinates (*a* and *b* values) respectively represent red–green. Namely, positive values are red, negative values are green, and yellow–blue means the larger the value indicates the yellower the color. The *b* values (19.9 and 21.9) and *L* values (48.2 and 48.3) of the leaves in the control at pH 5.70 and 6.70, showed that leaves grown in these treatments were yellower than others. However, the values of *a* were same among the treatments.

**Figure 3:**
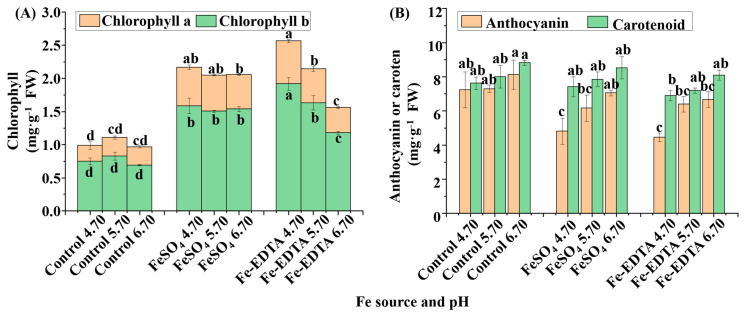
The contents of chlorophylls (**A**), carotenoid, and anthocyanin (**B**) in *H. macrophylla* after 60 days of cultivation; FW, fresh weight. Lowercase letters indicate significant differences calculated by Duncan’s multiple range test at *p* ≤ 0.05. Vertical bars indicate the standard error (*n* = 3). Lowercase letters indicate significant differences calculated by Duncan’s multiple range test at *p* ≤ 0.05.

The chlorophyll contents significantly changed in response to various Fe sources and pHs (Figure 3A), and the lowest chlorophyll a and b contents were found in the control group regardless of the pH. Similarly, the treatments with FeSO_4_ had little change among the different pHs, whereas, in the treatments with Fe-EDTA, the contents of chlorophyll a and b decreased with the increase of pH. Conversely, for the anthocyanin content, the lowest was observed in the treatments supplemented with FeSO_4_ and Fe-EDTA at pH 4.70, followed by the treatments supplemented with the same Fe sources at pH 5.70. However, there were same carotenoid content among Fe source treatments (Figure 3B).

Pearson’s correlation analysis revealed that the *L* value was positively correlated with the *b* value, and accordingly, the lightness of the leaves decreased as the leaves turned yellow. Similarly, there was a significantly positive correlation between the chlorophyll a and b contents. Furthermore, both chlorophyll a and b contents were negatively correlated with the *L* and *b* values, while the contents of chlorophyll b and anthocyanin were significantly negatively correlated (Table 5).

### 3.3. Analysis of the Stomata and Chlorophyll Fluorescence Parameters

**Figure 4:**
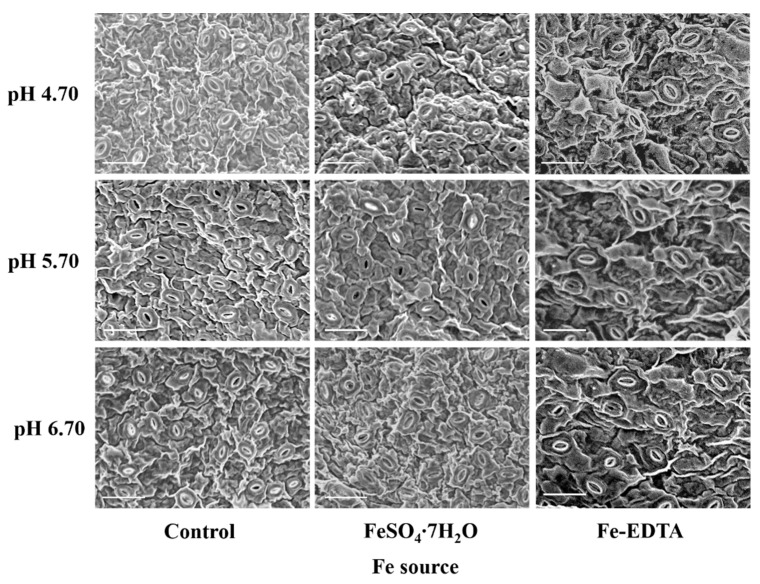
The SEM images of the stomata of *H. macrophylla* leaves after 60 days of culture. The bar indicates 50 μm.

**Figure 5:**
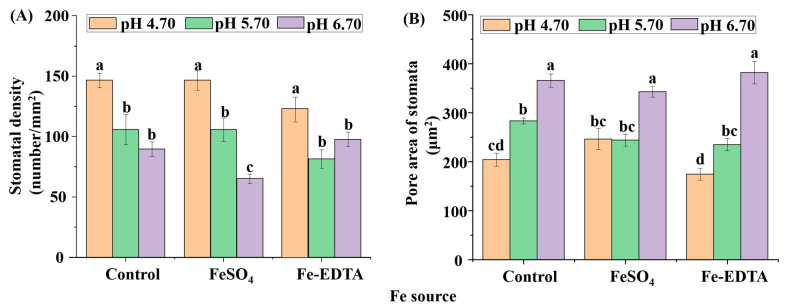
The stomatal density (**A**) and pore area of the stomata (**B**) of *H. macrophylla* leaves after 60 days of culture. Lowercase letters indicate significant differences calculated by Duncan’s multiple range test at *p* ≤ 0.05. Vertical bars indicate the standard error (*n* = 3).

Leaf photosynthetic efficiency is dependent on the light capture ability, as well limited by gas exchange of leaves, and stomatal development is closely related to photosynthesis. The stomata were observed to have few trichomes, and many fine grooves and ridges on the exposed surface of the leaves (Figure 4). There were no differences in the structure of the stomata among the different treatments in this study. The stomatal density in the visual field of the control and treatments with supplementary FeSO_4_ and Fe-EDTA at pH 4.70 were higher than that found in the other treatments. The lowest was observed in the treatment with FeSO_4_ at pH 6.70 (Figure 5A). The stomatal area was greatest at pH 6.70, while the treatments at pH 4.70 have smallest stomatal area regardless of Fe source (Figure 5B).

**Figure 6:**
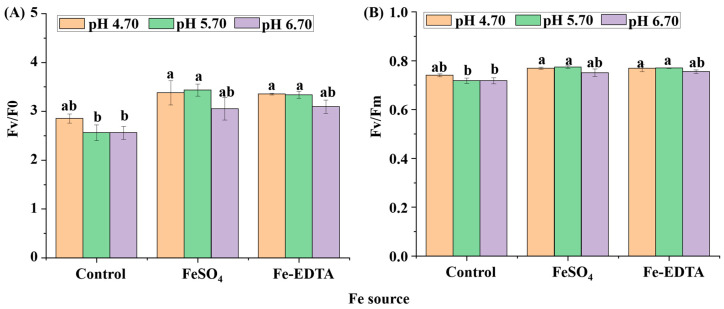
The chlorophyll fluorescence parameters Fv/F0 (**A**) and Fv/Fm (**B**) of *H. macrophylla* leaves after 60 days of culture. Lowercase letters indicate significant differences calculated by Duncan’s multiple range test at *p* ≤ 0.05. Vertical bars indicate the standard error (*n* = 3).

The chlorophyll fluorescence parameters of the maximum primary yield of PSII photochemistry (Fv/F0) and the maximum/potential quantum efficiency of PSII (Fv/Fm) are shown in Figure 6. The Fv/F0 values were significantly lower in the control group at pH 5.70 and 6.70. Similar results were found with the Fv/Fm values, where the minimum value was found in control treatments at pH 5.70 and 6.70. The Fe source and pH are directly related to the photosynthesis of *H. macrophylla*. The otherness of stomata also reflects the photosynthetic capacity of *H. macrophylla.*

### 3.4. Soluble Proteins, FCR, and Antioxidant Enzyme Activities

**Figure 7:**
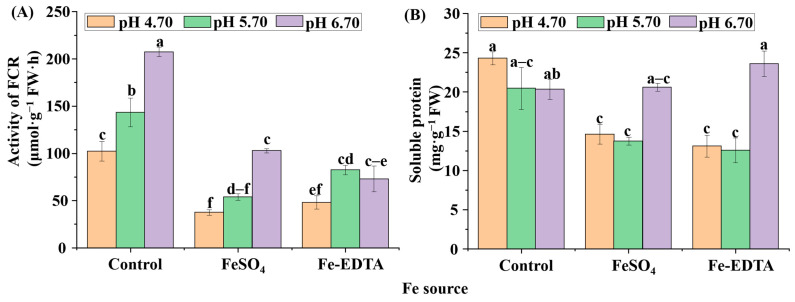
The ferric chelate reductase (FCR) activities (**A**) and soluble protein contents (**B**) in *H. macrophylla* after 60 days of culture. Lowercase letters indicate significant differences calculated by Duncan’s multiple range test at *p* ≤ 0.05. Vertical bars indicate the standard error (*n* = 3).

Protein is the structure of cells and the basis of life, including the activity of various enzymes. The soluble protein contents, and FCR activities were highly affected by the supplementary Fe source and pH (Figure 7). The FCR activities in the roots of the control group at pH 5.70 and 6.70 were significantly increased (Figure 7A), while the FCR activities decreased with supplementary FeSO_4_ and Fe-EDTA regardless of the pH. For the soluble protein content (Figure 7B), no significant differences were observed in the control group among the three pHs. The maximum soluble protein content was found with supplementary Fe-EDTA at pH 6.70, followed by supplementary FeSO_4_ at pH 6.70. There were no significant differences in the soluble protein content among treatments with supplementary FeSO_4_ and Fe-EDTA at pH 4.70 and 5.70.

**Figure 8:**
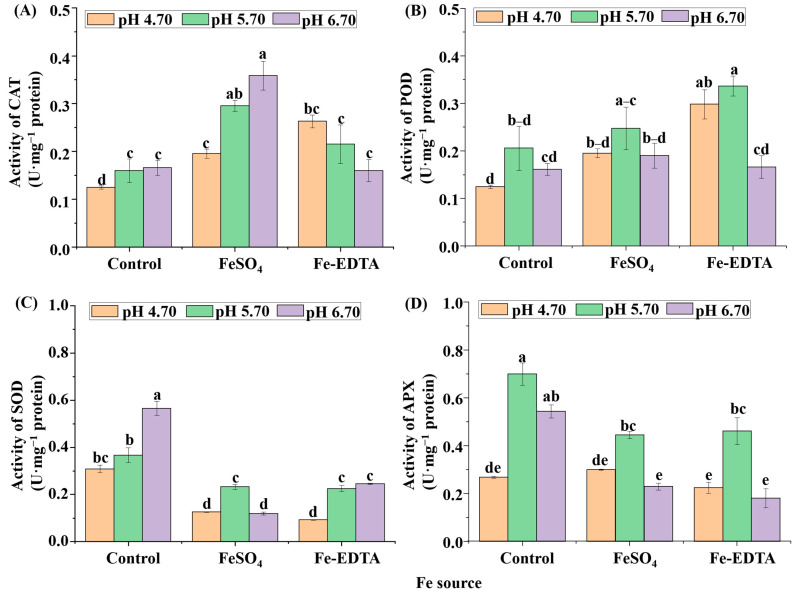
The activities of catalase (CAT) (**A**), guaiacol peroxidase (POD) (**B**), superoxide dismutase (SOD) (**C**), and ascorbate peroxidase (APX) (**D**) in *H. macrophylla* after 60 days of culture. Lowercase letters indicate significant differences calculated by Duncan’s multiple range test at *p* ≤ 0.05. Vertical bars indicate the standard error (*n* = 3).

According to the results in Figure 8, the CAT activity of control and supplementary FeSO_4_ increased with the pH, and reached the maximum value in the treatment supplemental FeSO_4_ at pH 6.70. Oppositely, under supplemental Fe-EDTA, CAT activity was decreased with the increased of pH. The results of SOD activity showed that the control group was significantly higher than the treatments supplemental Fe. Moreover, both POD and APX activities were the greatest when the pH was 5.70 regardless of Fe source (Figure 8D).

### 3.5. Micro- and Macronutrient Contents

The supply of Fe sources and different pHs directly affect the absorption of micro- and macronutrients of *H. macrophylla*. According to Table 6, there were significant differences in nutrient contents among all treatments. The tissue Fe content was highest in treatment supplemental Fe-EDTA at pH 4.70, which was 10 times the level found in the control group. In addition, the tissue contents of zinc (Zn), phosphorus (P), potassium (K), sulfur (S), and silicon (Si) of this treatment is highest among all treatments. However, the highest copper (Cu) and manganese (Mn) contents were observed in the control group. Following the results that lower pH can promote the absorption of Fe in the leaves of *H. macrophylla,* as well as increase the absorption of Zn, P, K, S, and Si, there was nevertheless a reduction of the absorption of Mn and Cu.

### 3.6. Quantitative Real-Time RT-PCR

**Figure 9:**
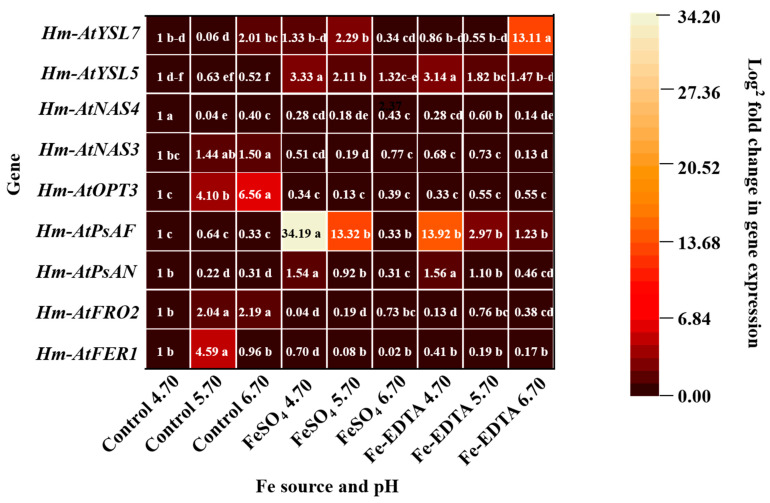
Expression profiles heat map of ferritin 1 (*FER1*), ferric reductase 2 (*FRO2*), photosystem I reaction center subunit N (*PsAN*), photosystem I subunit F (*PsAF*), oligopeptide transporters 3 (*OPT3*), nicotianamine synthase 3 (*NAS3*), *NAS4*, yellow stripe-like 5 (*YSL5*), and *YSL7* in *H. macrophylla* after 60 days of culture. The gene expression is presented with a scale of fold change calculated by 2^−^^ΔΔCT^. Lowercase letters indicate significant differences calculated by Duncan’s multiple range test at *p* ≤ 0.05.

Relative expressions of *FER1*, *FRO2*, *PsAN*, *PsAF*, *OPT3*, *NAS3*, *NAS4*, *YSL5*, and *YSL7* under different treatments showed significant differences (Figure 9). The expression of *Hm-AtFER1*, *Hm-AtFRO2*, *Hm-AtOPT3*, *Hm-AtNAS3*, and *Hm-AtNAS4* in the control groups were higher than that in the other treatments, and no significant differences were observed with the different supplementary Fe sources. Besides, the expressions of *Hm-AtPsAN*, *Hm-AtPsAF*, and *Hm-AtYSL5* were the highest with supplementary FeSO_4_ and Fe-EDTA at pH 4.70, and the *Hm-AtYSL7* showed the maximum expression with supplementary Fe-EDTA at pH 6.70.

## 4. Discussion

The concept of pH was derived from the ion product of water and was defined in terms of the hydrogen ion activity; namely, the negative logarithm of the hydrogen ion concentration [H^+^]. Researchers have found that the color of *H. macrophylla* sepals is very sensitive to the cultivation conditions and changes easily from red through purple, as the pH changes from 4.5 to 6.5 [41]. The pH influences the chemical solubility and the availability of essential nutrients for plants. Since H^+^ is a cation, it will compete with other cations for exchange sites. In alkaline soils, metal availability is often inhibited and causes micronutrient deficiencies in plants [42,43]. In general, *H. macrophylla* develops perfectly in acidic soils [44]. Moreover, a soil pH lower than 6.0 is beneficial to the formation of inflorescence and prolonged flowering duration, and increases plant growth [45,46]. In order to prevent Fe deficiency and assist in coloration, the suggested substrate pH range is 5.2 to 5.6 for greenhouse cultivation of *H. macrophylla* [47]. For Calcisol soil, only adjusting pH is not a good way to reduce the chlorosis, organic acids such as citric acid (CA), oxalic acid (OA), salicylic acid (SA), or humic complexes (HCs) combined with Fe chelate improved the quality of tomato [48]. In this study, the Fe accumulation in plantlets was promoted by a lower pH. On the contrary, the FCR activities were greatly enhanced at higher pH levels. Since the concentration of bio-available Fe is low, plants of strategy Ι usually up-regulated the FCR enzymes to take up the Fe in a controlled manner into the roots [49]. The enzymatic reduction of any Fe (III) complex must interact with the FCR to transfer the site of the electron [50]. However, the FCR reaction was most optimal at a pH of 5.0–5.5 [51]. A huge decrease (40 times) in the FCR reaction was observed in sugar beets (*Beta vulgaris* L.) at pH levels exceeding 6.2 [52]. Moreover, plants can reduce Fe uptake by rapidly decreasing FCR activity when Fe is excess. For example, the FCR activity in the rhizosphere of spinach and kale markedly decreased with increased supplemental Fe [53]. In this study, the Fe content was high in treatments with supplementary Fe-EDTA, as the Fe-EDTA reduced Fe to Fe^2+^ through the metal charge transfer reaction and the ligand is oxidatively degraded instead of being precipitated as the insoluble Fe oxides [54,55]. And the FCR reduction rates were slightly decreased due to the high stability of Fe-chelates [56]. Therefore, in treatments with supplementary Fe-EDTA, the FCR activities were lower than in treatments with supplementary FeSO_4_. However, for chlorosis plants, Fe concentration is more important compared with FCR activity, where foliar Fe-EDTA sprays were more effective than foliar FeSO_4_ sprays for correcting Fe deficiency in calibrachoa (*Calibrachoa hybrida*) [57].

Plants require different amounts of different nutrients. Macronutrients are needed in large amounts by plants, whereas micronutrients are essential to plants but only in small amounts. These nutrients interact with each other, in which deficiency or excess of these nutrients can upset this balance. Fe is indispensable for maintaining the metal homeostasis in plants. The researcher showed that the phytosiderophores also increase Zn, Cu, Mn, and Ni concentrations in wheat [58]. However, using Cu, Mn, and Zn as fertilizers for a long time is toxic to plants and may cause Fe deficiency. In this study, the content change of Fe also leads to this variation of Mn and Cu contents. Due to the Fe antagonistically interacting with other cationic micronutrients [59]. Differently, the Zn content does not seem to be significantly affected by supplementary Fe-EDTA, as Fe-EDTA exchanges and re-complexes to Zn-EDTA, resulting plants absorb Zn-chelate [60]. Similar result was found that both can increase the contents of Zn and Fe in plants by supplemental Zn and Fe fertilizer or only EDTA [61,62]. The leaf is the main sink of metal nutrients and chloroplasts allocate 80% of the Fe [63]. Furthermore, chloroplasts are a major site of the Fe function and ferritin storage for photosynthesis [49]. Fe influences photosynthesis through material structures and electron transport. Within the chloroplasts, 70~72% of the Fe is located in the thylakoids [64]. Thus, Fe deficiency will cause thylakoid growth to stagnate, and decrease the contents of chlorophyll and cytochrome *f* [63]. It is more objective to use the chlorophyll concentration than to use visual scores to test the Fe deficiency chlorosis [65]. In this study, we observed that the Fe deficiency led to greatly reduced chlorophyll contents, and the chlorophyll content decreased with the decrease in the Fe content. It was illustrated that the utilization of supplementary FeSO_4_ and Fe-EDTA could decrease chlorosis, which was in agreement with the findings in groundnut (*Arachis hypogaea* L.) and peppermint (*Mentha piperita* L.) [66,67]. Moreover, Fe deficiency caused reduction of the stomatal pores length, cuticular weight per unit surface, cuticular waxes, and stomatal turgor [68]. In this study, the treatments with supplementary Fe led to a lower stomatal density and higher stomatal size. In general, the stomatal density was negatively correlated with the stomatal size. Nevertheless, guard cells of stomata should be bigger than their nucleus [69], and according to the rule of “one-cell-spacing”, the stomata are separated by at least one epidermal cell in order to provide enough space for the other structures [70]. Besides, stomatal clustering will negatively impact the gas exchange rate [71]. Therefore, the plantlets with a lower number of larger stomata are more fit to maintain an appropriate photosynthetic rate [72]. It was worth noting that the shoot weight of pH 4.70 is higher than that of pH 6.70 with supplemental FeSO_4_, despite the leaf area and width of the latter were significantly higher than that of the former. The reason is that leaves with higher Fe content had better cuticle and epidermis development, and the biomass of petiol and stem were higher. In the photosynthetic electron chain, Fe acts as the ligature for PsbA and PsbD in PSII, PSII, and cytochrome b6/*f* (Cyt b6/*f*) in hemeprotein, and Cyt b6/*f* and ferredoxin in Fe–Sulfur [Fe–S] [73]. Therefore, chlorosis impairs the PSII while the PSΙ mostly unaffected [26]. As shown by the results, Fe deficiency decreased the Fv/F0 and Fv/Fm, findings which are consistent with those in pear and sugar beet [74,75]. Moreover, Fe deficiency decreased the activity of Rubisco, which regulates the Rubisco carboxylation efficiency [76], it has been reported that there are a high correlations among apparent Rubisco carboxylation efficiencies, leaf absorptances, and PSII efficiencies [77]. To sum up, the evidence is that Fe deficiency reduces photosynthesis by decreased chlorophyll, gas exchange, and activities of PS. However, more research is still needed.

**Figure 10:**
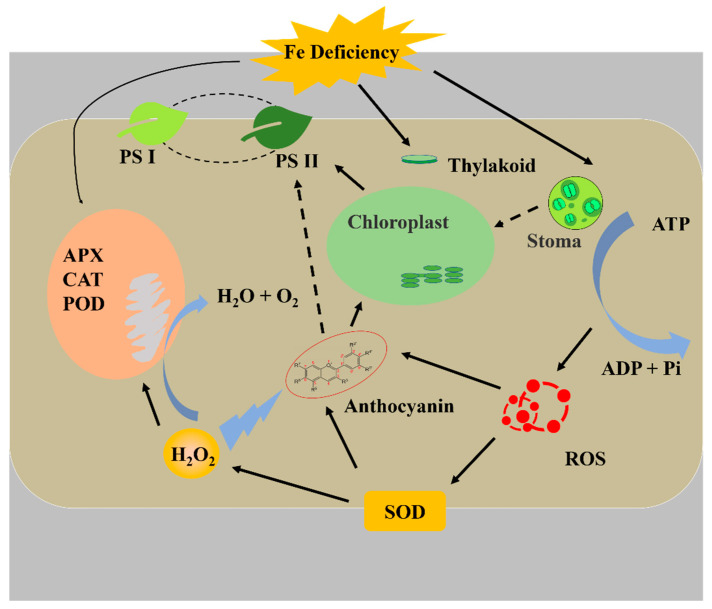
The relational model of Fe deficiency with antioxidant system and photosynthesis.

The relationships between the leaf color, and the contents of chlorophyll, anthocyanin, and carotenoid are noteworthy. Supplementary Fe increased the anthocyanin contents in leaves. Similarly, it was reported that Fe regulates the anthocyanin biosynthetic pathway and Fe supply increased the total anthocyanins content in *Vitis vinifera* [78]. However, it has not yet been elucidated if Fe has a direct or indirect effect on anthocyanins and their biosynthesis pathway. Although the accumulation of carotenoids and flavonoids mainly affects the flower color, they also influence the leaf color. Anthocyanin has a negative effect on photosynthesis, which could absorb more green light and accelerate the degradation of thylakoids, which causes a decrease in the photosynthetic rate [79]. Another suggestion is that anthocyanin acts as an antioxidant scavenging free radical and reduces the electron chain in photosynthesis [80]. The process is similar to the xanthophyll cycle. It has been reported that Fe deficient increased zeaxanthin to form light protection in pear (*Pyrus communis*) [74]. However, there is no evidence that anthocyanin was prominently photo-inhibitory or antioxidant in this study. It is can be determined that changes in the anthocyanins often indicate the variety of the antioxidant system. The SOD defends against oxidation and produces H_2_O_2_, then the H_2_O_2_ is scavenged by APX, CAT, and POD. It is reported that a higher SOD amount is found in purple leaves, which contain more anthocyanins, and H_2_O_2_ can induce the signal for anthocyanin biosynthesis [79,81]. In addition, APX, CAT, and POD act as the hemeproteins and their activities are affected both by the Fe content and ROS [82]. It has been reported that POD is more sensitive to H_2_O_2_ than CAT [83]; therefore, in this study, the activity of POD increased under pH 5.70, and similar results were observed with the activity of APX, it is speculated that the APX has a higher sensitivity to H_2_O_2_. In brief, as shown in Figure 10, Fe deficiency can directly reduce chlorophyll synthesis and affect plant growth. Furthermore, Fe deficiency increases the ROS content, which induces the antioxidant system, while insufficient antioxidant hemeproteins exacerbate the damage to plantlets.

The control of the Fe-homeostasis is handled at the genetic level. First, the reduction of the ferric chelates by ferric reductase is an obligatory step in Strategy Ι plants. Overexpression of *FRO2* in *Arabidopsis thaliana* was observed to correspond with a significant increase in the tissue content of Fe [84]. Meanwhile, the *AtFRO2* is controlled by a transcriptional factor *FER*, reported to be the spontaneous mutant *T3238fer*, which is unable to activate Fe-deficiency responses under low Fe conditions, which includes but is not limited to the expression of *FRO* and the activity of ferric chelate reductase [85]. In this study, Fe deficiency upregulated the expression of *Hm-AtFRO2* and *Hm-AtFER1*, whereas the *Hm-AtFER1* in the control at pH 5.70 was significantly higher than that in the control group at the other two pH levels, which is speculated to be related with the underlying FER regulatory pathway in strategy Ι. The detailed mechanisms involved remain to be revealed. Second, once inside the roots, Fe is transported over long distances via the veins to the tissues by transpiration. Previous researchers have found that aerial part of the plant has a key role in the distribution and regulation of Fe, in both strategy I and strategy II plants, intracellular delivery of Fe and trafficking within the plant is dependent on NA, which chelates and transports micronutrient metal ions. The lack of NA would decrease the mobility and availability of Fe, as well as form precipitation in the chloroplast and phloem sap [22]. The Fe-NA complexes are transferred from the xylem to the neighboring cells through the YSL proteins in *Arabidopsis* [86]. These are regulated by *NAS* and *YSL*. Analysis of the Fe-sufficient and Fe-deficient transcriptome in *Arabidopsis* revealed that NAS4 was strongly Fe-regulated in leaves [22]. Differently, in this study, *Hm-AtNAS3* and *Hm-AtNAS5* were respectively upregulated by Fe deficiency and supplementary Fe. It is speculated that these genes are also regulated by Cu and Zn [87], as the YSL protein is able to transport metal-NA complexes and NA has a high affinity for the two metals. The YSL transporters belong to the OPT family. Under Fe deficiency, the *YSL5* was upregulated nearly twice in order to improve the Fe nutrient condition in apple [88]. Similar results were found with *YSL7* in peanut. The *OPT3*, which also belongs to the OPT family, is preferentially expressed in the phloem cells during Fe deficiency [89]. The *OPT3* functions in the phloem-mediated Fe redistribution, suggesting that *OPT3* transfers the Fe out of the leaves [90]. Therefore, the increased expression of *Hm-AtOPT3* in the control group exacerbated the Fe deficiency in the leaves. It is speculated that the shoots feedback the Fe deficiency signals to the roots through phloem. Similar reports found after foliar spray Fe, leaves of *A. thaliana* transmit signals to roots via the Fe-peptide transport regulated. Besides, Fe content of the phloem in Fe deficiency leaves was lower than in Fe supplemental leaves in castor oil (*Ricinus communis* L.) and dwarf bean (*Phaseolus vulgris* L.) [91,92], and the unallocated Fe is stored by the leaves [93]. The chlorosis did the most damage to the PSII, and it has been documented that PSII is more sensitive to Fe deficiency [26,94,95]. Another view is PSI is a prime target for Fe deficiency due to its high Fe demand [96]. In this study, the results showed that there was the huge upregulated expression of *Hm-AtPsAN* and *Hm-AtPsAF* with supplementary Fe. PsAN and PsAF proteins are connected with PSI and plastocyanin (Pc), where the function of the latter is the transfer of electrons from Cyt b6/*f* to the PSI complex in the thylakoid lumen [97,98]. Interestingly, reported found in the thylakoid membranes of Fe deficiency leaves, core proteins of PSI (PsaA) declined drastically, while the core proteins of PSII (PsbA) remained stable relatively [99,100]. Furthermore, the IsiA protein, which is homologous to the inner antenna protein of PSII (PsbC), and expressed specifically under Fe deficiency. It is involved in the state transitions of light sharing between two photosystems, which needs further study.

## 5. Conclusions

Our results showed that the growth and development of in vitro *H. macrophylla* was affected by supplementary Fe. The Fe deficiency induced chlorosis and decreased the biomass of plants. Application of supplementary Fe enhanced the number of leaves and roots, fresh/dry shoot and root weights, and the chlorophyll contents, and increased the photosynthesis. Supplementary Fe also changed the macro- and micronutrient contents, especially the Fe uptake in *H. macrophylla*. The *H. macrophylla* had the best Fe absorption at pH 4.70. Among all the Fe sources and medium pH studied, Fe-EDTA at pH 4.70 was found to be the most effective in promoting the growth and development of *H. macrophylla* in vitro.

## Figures and Tables

**Table 1:** Genes of *Arabidopsis thaliana* locus number and name used in this study.

Full Name	Abbreviation	Locus ID
*AtFerritin 1*	*AtFER1*	AT5G01600
*AtFerric reductase oxidase 2*	*AtFRO2*	AT1G01580
*AtPhotosystem I subunit N*	*AtPsAN*	AT5G64040
*AtPhotosystem I subunit F*	*AtPsAF*	AT1G31330
*AtOligopeptide transporters 3*	*AtOPT3*	AT4G16370
*AtNicotianamine synthase 3*	*AtNAS4*	AT1G09240.1
*AtNicotianamine synthase 4*	*AtNAS3*	AT1G09240.2
*AtYellow stripe-like 5*	*AtYSL5*	AT3G17650
*AtYellow stripe-like 7*	*AtYSL7*	AT1G65730

**Table 2:** List of qPCR primers used in this study.

Gene Name	Forward Primers (5’ to 3’)	Reverse Primers (5’ to 3’)
*Hm-AtFER1*	ATGCTTTATATGCAATGGAGTTGGC	CTGGTCGAAATGCCAAACTCCG
*Hm-AtFRO2*	CTTCCTTCCGACACTATGGAGC	CAGAAGAAGAAAGCCTCTGGTG
*Hm-AtPsAN*	TCATTGACGACTACCTGGAAAAGAG	CCAGAAAACATTGGAACCGCAC
*Hm-AtPsAF*	ACTTGATAGTGAACGGAGACCAGCG	AGAAACTCTCTGTAGGCAGCAACGG
*Hm-AtOPT3*	GCACTTCCAGGCTATCTATTCCC	TCCAGTAACAAACAGGGACGATG
*Hm-AtNAS4*	AAAATCTGTGGTGAGGCTGAAG	TTGAGATGGGAAGAGGCAAGAAC
*Hm-AtNAS3*	CGCTTCTCTTCTGGTTTCCTCTG	AAATAGACAAAACCTCGAACCCCTG
*Hm-AtYSL5*	TATCAACAGCTTTCACACCCCTC	ACGGACATATCATTCCAACACCAAC
*Hm-AtYSL7*	CATTTGGGTTGGATGATTGGATTTC	TTGAAAGAAACCCCACAAGAAACTG
*Hmactin*	GCCTGCCATGTATGTTGCCATC	CGGAATCCAGCACAATACCAGTTG

**Table 3:** The growth parameters of *H. macrophylla* after 60 days of culture.

Fe Source (A)	pH (B)	Leaf				Shoot			Root				Stem Diameter (mm)
		Number	Area (cm^2^)	Length (cm)	Width (cm)	Length (cm)	Fresh Weight (mg)	Dry Weight (mg)	Number	Length (cm)	Fresh Weight (mg)	Dry Weight (mg)	
Control	4.70	9 b–d ^z^	10.3 a–c	2.1 bc	1.9 a	3.8 a	300.4 bc	57.0 a–c	3 d	5.3 b–d	51.2 cd	3.0 b–d	2.4 ab
	5.70	7 cd	4.9 bc	1.9 c	1.3 a	4.7 a	179.7 c	34.6 c	3 d	2.7 e	21.7 d	0.9 d	2.7 ab
	6.70	6 d	3.6 c	2.1 bc	1.3 a	4.4 a	180.0 c	42.4 bc	3 d	5.1 cd	46.3 cd	1.7 cd	3.0 a
FeSO_4_ H_2_O	4.70	14 a	13.2 ab	3.2 a	1.7 a	5.6 a	757.2 a	86.9 a	8 a	7.0 a	117.9 a	6.9 a	2.1 ab
	5.70	12 ab	9.7 a–c	2.8 ab	1.5 a	4.4 a	377.6 bc	56.9 a–c	6 a–c	5.7 a–d	59.5 b–d	4.1 a–c	2.5 a
	6.70	10 bc	14.6 a	2.7 a–c	2.1 a	4.6 a	394.6 bc	61.9 a–c	7 ab	6.7 a–c	88.9 a–c	5.2 ab	1.7 ac
Fe-EDTA	4.70	12 ab	12.1 a–c	2.6 a–c	1.7 a	4.6 a	424.1 b	69.9 ab	6 a–c	6.9 ab	107.0 ab	6.9 a	3.0 a
	5.70	11 ab	15.0 a	3.2 a	1.8 a	4.7 a	309.1 bc	51.4 bc	5 bc	5.2 cd	60.9 b–d	3.2 bc	2.1 ab
	6.70	9 b–d	9.1 a–c	2.6 a–c	1.7 a	5.1 a	490.1 b	63.3 a–c	5 bc	4.8 d	50.5 cd	4.4 a–c	2.8 a
F-test ^y^	A	***	*	**	NS	NS	***	NS	***	***	*	**	**
	B	*	NS	NS	NS	NS	NS	*	NS	NS	NS	NS	NS
	A × B	NS	NS	NS	NS	NS	**	**	*	**	**	NS	**

^z^ Lowercase letters indicate significant differences calculated by Duncan’s multiple range test at *p* ≤ 0.05; ^y^ NS, *, **, and *** represent non-significant or significant at *p* ≤ 0.05, 0.01, and 0.001, respectively.

**Table 4:** The colors of *H. macrophylla* leaves after 60 days of cultivation.

Fe Source (A)	pH (B)	L*	*a*	*b*
Control	4.70	44.7 ab ^z^	−15.1 a	15.7 ab
	5.70	48.2 a	−16.1 a	19.9 a
	6.70	48.3 a	−11.0 a	21.9 a
FeSO_4_	4.70	41.3 ab	−15.5 a	6.3 bc
	5.70	37.7 bc	−18.0 a	11.5 bc
	6.70	37.8 bc	−14.1 a	10.0 bc
Fe-EDTA	4.70	37.7 bc	−14.6 a	3.0 c
	5.70	30.8 c	−11.0 a	3.6 bc
	6.70	37.8 bc	−12.0 a	8.8 bc
F-test ^y^	A	***	NS	*
	B	NS	NS	NS
	A × B	***	NS	NS

* *L*, lightness; *a*, red (+)/green (−) color attribute; *b*, yellow (+)/blue (−) color attribute values. ^z^ Mean separation within columns for each cultivar by Duncan’s multiple range test at *p* = 0.05. ^y^ NS, *, ***, not significant or significant at *p* = 0.05, or 0.001, respectively.

**Table 5:** Correlation analysis between the leaf color and leaf pigment contents in this study.

Correlation Coefficient	*a* *	*b* *	Chlorophyll *a*	Chlorophyll *b*	Anthocyanin	Carotenoid
*L* *	−0.159	0.646 **	−0.580 **	−0.511 **	0.010	0.137
*a* *		−0.244	−0.096	−0.095	0.028	0.248
*b* *			−0.501 **	−0.425 *	0.039	0.040
Chlorophyll *a*				0.964 **	−0.370	−0.256
Chlorophyll *b*					−0.411 *	−0.160
Anthocyanin						−0.038

* *L*, lightness; *a*, red (+)/green (−) color attribute; *b*, yellow (+)/blue (−) color attribute values. **, * correlation is significant at *p <* 0.01, 0.05, respectively.

**Table 6:** Mineral contents in *H. macrophylla* after 60 days of culture.

Fe Source (A)	pH (B)	Micronutrient (mg·g^−1^ DW)				Macronutrient (mg·g^−1^ DW)					Si (mg·g^−1^ DW)
		Fe	Mn	Zn	Cu	P	K	Ca	S	Mg	
Control	4.70	0.13 f ^z^	0.20 b	0.06 c	0.09 c	9.63 e	18.61 f	15.02 e	26.34 c	2.84 e	0.13 f
	5.70	0.10 f	0.20 b	0.04 d	0.10 b	10.08 e	23.27 e	33.16 d	21.95 d	3.23 e	0.12 f
	6.70	0.06 g	0.47 a	0.04 d	0.28 a	3.17 f	6.69 g	4.80 f	10.03 g	2.07 f	0.07 g
FeSO_4_	4.70	0.36 c	0.10 e	0.06 c	0.02 fg	27.88 b	80.37 b	161.29 a	37.96 b	6.59 c	0.48 b
	5.70	0.25 de	0.19 b	0.10 b	0.04 e	18.21 c	39.89 d	94.00 b	25.25 c	12.63 a	0.35 c
	6.70	0.23 e	0.14 d	0.07 c	0.07 d	10.31 e	21.13 ef	14.85 e	12.32 f	3.45 e	0.14 f
Fe-EDTA	4.70	1.12 a	0.07 f	0.18 a	0.02 fg	52.51 a	134.80 a	74.21 c	63.45 a	6.43 cd	0.80 a
	5.70	0.49 b	0.10 e	0.09 b	0.02 fg	17.53 c	47.04 c	33.08 d	14.12 e	10.70 b	0.23 d
	6.70	0.26 d	0.17 c	0.07 c	0.03 f	14.67 d	37.95 d	37.71 d	12.77 f	5.88 d	0.19 e
F-test ^y^	A	***	***	***	***	***	***	***	***	***	***
	B	***	***	***	***	***	***	***	***	***	***
	A × B	***	***	***	***	***	***	***	***	***	***

^z^ Lowercase letters indicate significant differences calculated by Duncan’s multiple range test at *p* ≤ 0.05; ^y^ *** represents significant at *p* ≤ 0.001.

## Data Availability

Not applicable.
